# Supplementary material for: Selective and Controllable Trapping of Single Proteins in Nanopores Using Reversible Covalent Bonds
Source: ACS Nano. 2025 Dec 21;20(1):879–91. doi: 10.1021/acsnano.5c16000 (PMC12810480; doi:10.1021/acsnano.5c16000)
Supplement: Supplementary file 1 [file nn5c16000_si_001.pdf]

Supplementary Information

Selective and Controllable Trapping of Single Proteins in Nanopores using Reversible Covalent Bonds

Yuanjie Li, [1] Saurabh Awasthi, [1, 2] Peng Liu, [1] Anna D. Protopopova,[1] Michael Mayer[1*]

[1] Adolphe Merkle Institute, University of Fribourg, Chemin des Verdiers 4, CH-1700 Fribourg, Switzerland

[2] Department of Biotechnology, National Institute of Pharmaceutical Education and Research, Raebareli (NIPER-R), Lucknow-226002, Uttar Pradesh, India

*Corresponding Author Email: michael.mayer@unifr.ch

Supplementary Note S1: Characterization of polymer coatings for nanopores

In order to estimate the volume and shape of single proteins from resistive pulse recordings, the electric field inside the nanopore must be as uniform as possible.[1] To this end, the shape of the nanopore should approach as well as possible an ideal cylinder. The dimensions of a perfectly cylindrical nanopore can be determined from the ionic current using Equation S1:

$$G = \frac{\sigma}{\frac{l_p}{\pi {r_p}^2} + \frac{1}{2r_p}} \quad (S1)$$

Here, G (Ω^{-1}) is the conductrance of the nanopore measured from the *I-V* curve, σ ($\Omega^{-1}m^{-1}$) is the conductivity of the electrolyte solution, l_p (m) is the length of the nanopore channel, and r_p (m) is the radius of the nanopore.

After coating the nanopore with a polymer, both the length and radius change by the effective thickness of the coating layer, l_c. We determined the thickness of the coating experimentally from the measured conductance, using Equation S2:

$$G = \frac{\sigma}{\frac{l_p + 2 * l_c}{\pi(r_p - l_c)^2} + \frac{1}{2(r_p - l_c)}} \quad (S2)$$

Supplementary Note S2: Glucose binding fitted with a dual-site Langmuir model

Glucose contains two vicinal diol motifs (C1–C2 and C5–C6)[2] that can form reversible boronate ester bonds with PBA groups on the PAcrAm-g-PEG-PBA coating. As a result, the binding process is better described by a two-site Langmuir isotherm:

$$y = \frac{B_1 x}{k_1 + x} + \frac{B_2 x}{k_2 + x} \quad (S3)$$

Here, B_1 and B_2 represent the maximum binding capacities of the two binding modes, while k_1 and k_2 represent their respective dissociation constants, reflecting binding affinities.

Supplementary Note S3: Calculation of dissociation rate constant for the reaction between PBA functional group and gHSA

When a protein with a net charge is trapped by a reversible covalent bond, the bond will be affected by the electrophoretic force acting on the trapped protein in the strong electric field inside the nanopore.[3] Therefore, the change in Gibbs Free Energy, ΔG, for bond breaking is directly influenced by the external force.[4] The rate constant for the dissociation reaction, k, is hence related to the Arrhenius equation in the following way:

$$k = Ae^{\Delta G - Eq\alpha} \quad (S4)$$

Here, A is the pre-exponential (Arrhenius) factor, ΔG is the Gibbs energy of activation in the absence of an external electric field, E is an external electric field, q is the net charge of the protein with its linkers, and α is a coefficient related to the length of nanopore channels.[5]

Taking the natural logarithm of **Equation S4** yields a linear relationship (**Equation S5**), which makes it possible to estimate the dissociation rate constant k_0 at 0 mV applied potential through least-squares fitting of the experimental data shown in **Figure 4C**:

$$lnk = lnk_0 - Eq\alpha \quad (S5)$$

With regard to the number of glycation sites on proteins and the relationship between rotational diffusion time of bound and unbound proteins compared to the translocation time of unbound proteins, we note that glycation can typically occur on the side chains of lysine and arginine amino acids. According to the specifications provided by the supplier, the glycated human serum albumin (HSA) used in our experiments has a molar ratio of sugar to HSA ranging from 1:1 to 5:1 (i.e., one to five glycations per HSA protein). In general, most proteins likely contain multiple accessible side chains that could be glycated, depending on the protein, its size, and the conditions in which the protein was expressed, stored and purified that may or may not favor glycation (for instance, whether glucose, fructose or other molecules with vicinal diols were present at significant concentrations during any of these steps). During the translocation of proteins, each glycated side chain will have a certain probability to form a reversible covalent bond with PBA groups, depending on the surface density and accessibility of both reaction partners, the reaction conditions, the residence time in the pore, and the relative orientation of the groups towards each other during their collision.

Since we observed off rates that compared well with those reported for the dissociation of single bonds between PBA groups and vicinal diols, and since we observed a significant fraction of free translocation events, we hypothesize that most protein translocations formed either zero reversible covalent bonds (i.e., free translocations with short translocation times below 0.4 ms) or one reversible covalent bond. In rare instances, two or more reversible bonds

may have formed, explaining the few exceptionally long residence times. Thus, for the majority of long trapping events, we expect that the proteins were attached to the nanopore wall by one PBA-diol anchor. Since these anchors contain multiple sigma bonds, rotation of trapped proteins around those bonds is possible, albeit presumably with a reduced rotational diffusion coefficient due to the attachment to the anchor. In addition, Figure 1 illustrates that the anchor was relatively long and flexible, with two segments of PEG units, making it possible to sample various orientations of the proteins through flopping motions of the anchored protein.

The estimated rotational diffusion time for a protein like HSA in solution is on the order of 100 ns, and the time for non-bound diffusive translocation through a 30 nm pore is on the order of microseconds. This comparison indicates that during translocation, the rotation of the protein is significantly faster than its translation (see Haughtaling et al[8]).

Supplementary Note S4: Calculation of the frequency of events

Since the frequency of resistive pulses may vary over time, we used instantaneous frequency to compute the frequency of long and short events of individual resistive pulses:

$$f_i = \frac{2}{t_{i+1} - t_{i-1}} \quad (S6)$$

Where f_i is the frequency of ith event, and t_{i-1} and t_{i+1} are the start times of the $(i-1)^{th}$ and $(i+1)^{th}$ events. The total event frequency was determined as a mean value across all instantaneous frequencies.

Supplementary Note S5: Determination of the effective nanopore length using resistive pulses from a spherical protein

We determined the effective nanopore length, $l_{p,eff}$, with the spherical protein streptavidin whose molecular shape can be approximated as a sphere with a length-to-diameter ratio, $m = 1$, electrical shape factor, $\gamma = 1.5$, and volume, $V_{SA} = 101$ nm^3.[1, 6]

To characterize each nanopore, we recorded approximately one hundred resistive pulses with streptavidin ($^{\Delta I}/_{I_0}$). For quantitative analysis, we used the nanopore diameter d_p as provided by TEM image and derived the effective length of the nanopores, $l_{p,eff}$, from Equation 1 as follows:

$$l_{p,eff} = \frac{6V_{SA}I_0}{\pi d_p^2 \Delta I} - 0.8d_p \quad (S7)$$

Supplementary Note S6: Estimation of protein shape and volume

We detected resistive pulses by the so-called two sliding windows algorithm, which has been developed from the threshold detection algorithm.[7] We use the analysis of individual resistive pulses to calculate the shape and volume of the proteins based on previous work from our group by Houghtaling et al.[8] The rotation of non-spherical proteins while they move through the nanopore changes their orientation relative to the electric field and modulates the recorded ionic current blockade, ΔI as a function of the electrical shape factor, γ. The probability density function of ΔI can be described as **Equation S8** for oblate and **Equation S9** for prolate ellipsoids:

$$P(\Delta I_\gamma) = \frac{1}{A}\cosh\left(\frac{E\mu\left(\sqrt{\frac{\Delta I - \Delta I_{min}}{\Delta I_{max} - \Delta I_{min}}}\right)}{k_B T}\right)\frac{1}{\pi\sqrt{(\Delta I - \Delta I_{min})\,(\Delta I_{max} - \Delta I)}} \quad (S8)$$

$$P(\Delta I_\gamma) = \frac{1}{A}\cosh\left(\frac{E\mu\left(\sqrt{\frac{\Delta I - \Delta I_{max}}{\Delta I_{min} - \Delta I_{max}}}\right)}{k_B T}\right)\frac{1}{\pi\sqrt{(\Delta I - \Delta I_{min})\,(\Delta I_{max} - \Delta I)}} \quad (S9)$$

Here, $P(\Delta I_\gamma)$ is the probability density distribution of ΔI, which is dependent on the orientation-dependent electrical shape factor, γ. E is the electric field, μ is the dipole moment, A is a normalization constant for integration, and ΔI_{min} and ΔI_{max} are the minimum and maximum of ionic current from the respective resistive pulses as a response to the rotation of a single protein during its translocation.

This probability distribution does not account for noise in the current recording, so we convolve this U-shaped distribution with a standard Gaussian noise distribution function as shown in **Equation S10**:

$$P(\Delta I_\sigma) = \frac{1}{\sqrt{2\pi\sigma^2}} e^{-\frac{\Delta I_\sigma{}^2}{2\sigma^2}} \quad (S10)$$

To this end we convolve the U-shaped probability distribution with standard Gaussian noise distribution to describe the probability of the blockade of current, ΔI.

$$P(\Delta I) = P(\Delta I_s) * P(\Delta I_\sigma) \quad (S11)$$

To carry out this convolution, we employed the *lsqcurvefit* function by MATLAB with four parameters that we initialized to follows: the protein permanent dipole moment, μ = 550 D, ΔI_{min} and ΔI_{max} are 5th and 95th percentiles of the current blockade, the σ is greater than, or equal to, the standard deviation of baseline noise. The length-to-diameter ratio, m, of the proteins was calculated by the following equations.

For an oblate-shaped protein:

$$\frac{\Delta I_{max}}{\Delta I_{min}} = \left(\frac{m \cdot \cos^{-1}(m)}{(1-m^2)^{1.5}} - \frac{m^2}{1-m^2}\right)^{-1} - 0.5 \quad (S12)$$

For a prolate-shaped protein:

$$\frac{\Delta I_{min}}{\Delta I_{max}} = \left(\frac{m^2}{m^2-1} - \frac{m \cdot \cos^{-1}(m)}{(m^2-1)^{1.5}}\right)^{-1} - 0.5 \quad (S13)$$

Here the ΔI_{min}, and ΔI_{max} are determined by fitting **Equations S12** or **S13** to the probability density distribution function of ΔI. $\gamma_{\parallel}$ describes the values of electrical shape factor of the

ellipsoid when its singleton axis is aligned parallel to the electric field, ***E***. $\gamma_{\perp}$ describes the singleton axis aligned perpendicular to the electric field, ***E***. **Equations S14** or **S15** describe the calculation of $\gamma_{\parallel}$ and $\gamma_{\perp}$ for oblate and prolate, respectively.

$$\gamma_{\parallel} = \frac{\Delta I_{max}}{\Delta I_{min}} + 0.5 \quad (S14)$$

$$\gamma_{\perp} = \frac{\Delta I_{min}}{\Delta I_{max}} + 0.5 \quad (S15)$$

Perfect spheres always have a constant electrical shape factor of 1.5. We determine the volume of protein, *V*, by the theory of Maxwell's derivation for translocating particle trace[6]:

$$\frac{\Delta I}{I_0} = -\frac{4V\gamma}{\pi d_p^2 \left(l_p + 0.8 d_p\right)} \left(\frac{1}{1 - 0.8 \left(\frac{d_m}{d_p}\right)^3} \right) \quad (S16)$$

Here, V is the volume of the protein, γ is the electrical shape factor, d_p is the diameter of the nanopore, l_p is the length of the nanopore channel, and d_m is the diameter of the protein. To enable rotation of proteins during their translocation, we used nanopores with diameters that were at least twice the longest dimension of all tested proteins.

With regard to the ability of the approach to discriminate between different volumes, we note that for the proteins represented in **Figure 6**, the average uncertainty of the determined volumes is 140 nm^3. An exception is Thyroglobulin, which has a significantly larger uncertainty, possibly due to its dimeric nature and large, elongated shape. However, if the goal is to discriminate between different proteins, we suggest employing information on the size and shape of the proteins for discrimination (and possibly additional features from each resistive pulse, such as skewness of the $\Delta I/I_0$ distribution). In this case, the prolonged residence time of proteins will help to improve the classification accuracy.

With regard to selective detection of proteins in serum samples, the large size distribution and large fraction of human serum albumin in serum may obscure resistive pulses from other

proteins of interest, such as protein biomarkers. Therefore, for most applications in serum samples, a purification and possibly a concentration step, such as a "pull-down" reaction with an antibody specific to the target protein, may be necessary. On the other hand, if the goal were to determine the total concentration of glycated proteins in serum samples, then a dilution of the serum sample, combined with an appropriate calibration, may make such a determination possible using the approach presented.

Supplementary Table S1. Theoretically estimated net charge and corresponding theoretically predicted dwell time of translocation of gHSA as a function of pH.

pH	Theoretical charge (e^-)[a]	Theoretical dwell time (μs)[b]
5.0	+4.0	5.6
6.0	-2.9	5.1
6.5	-6.0	4.0
7.0	-8.0	3.2
7.5	-15.0	2.1
8.0	-16.0	2
8.5	-18.0	1.8

(a) Net charge of human serum albumin is calculated from the crystal structure using APBS&PDB2PQR web service (https://server.poissonboltzmann.org/). This calculation did not account for glycation.

(b) Theoretical dwell time represents the most probable values from the first passage time distribution of free translocation.[9] Using the following equation:

$$PDF = \frac{l}{\sqrt{4\pi D t^3}} e^{\frac{-\left(l-\frac{qDE}{k_B T}t\right)^2}{4Dt}}$$

with the following parameters: length of nanopore, l: 30 nm, electrical field, E: $3.3e^6$ V/m, diffusion coefficient of proteins, D: $1e^{-11}$ m^2/s.

Supplementary Table S2. Comparison of molecular weight, isoelectric point, length-to-diameter ratio, and excluded volume of four test proteins.

Protein	Molecular Weight (kDa) [a]	pI [b]	Length-to-diameter ratio [c]	Volume (nm^3) [d]	Volume (nm^3) [e]
gHSA	66.5	4.7	0.65	147	80
HbA1c	64.5	6.9	0.81	133	78
IgG	150	7.3	0.46	332	190
Tg	660	4.5	1.86	1247	790

(a) Obtained from the protein data bank (https://www.rcsb.org).

(b) Estimated from the amino sequence using web service (https://www.protpi.ch/Calculator/ProteinTool).

(c) Calculated from the crystal structure using Minimum Volume Enclosing Ellipsoids (MVEE) fitting.[10]

(d) Calculated from the crystal structure using Solvent Accessible Volume (SAV) with a diameter of probe of 0.28 nm.[11]

(e) Calculated from the molecular weight[12] *with following equation:*

$$V = \frac{4}{3}\pi\left(0.066\sqrt[3]{MV}\right)^3$$

Supplementary Table S3. Accuracy of volume and shape estimation of individual proteins using resistive pulse data from short and long resistive pulses.

	gHSA	HbA1c	IgG	Tg	Average absolute value
Ref. *V*	147	133	332	1248	
150 µs< t_d <400 µs	139 ± 27	93 ± 14	180 ± 205	1006 ± 983	
Percent deviation (%)	-5.3	-30.1	-45.6	-19.4	25.1 ± 42.3
t_d > 400 µs	140 ± 22	100 ± 7	412 ± 154	1323 ± 761	
Percent deviation (%)	-4.5	-25.0	+24.1	+6.8	15.1 ± 31.8
Ref. *m*	0.65	0.81	0.46	1.86	
150 µs< t_d <400 µs	0.62 ± 0.19	0.72 ± 0.17	0.57 ± 0.28	2.45 ± 1.35	
Percent deviation (%)	-4.6	-11.1	+23.9	+31.7	17.8 ± 45.9
t_d > 400 µs	0.62 ± 0.12	0.74 ± 0.1	0.55 ± 0.24	1.75 ± 1.34	
Percent deviation (%)	-4.6	-7.4	+19.6	-5.9	9.4 ± 38.7

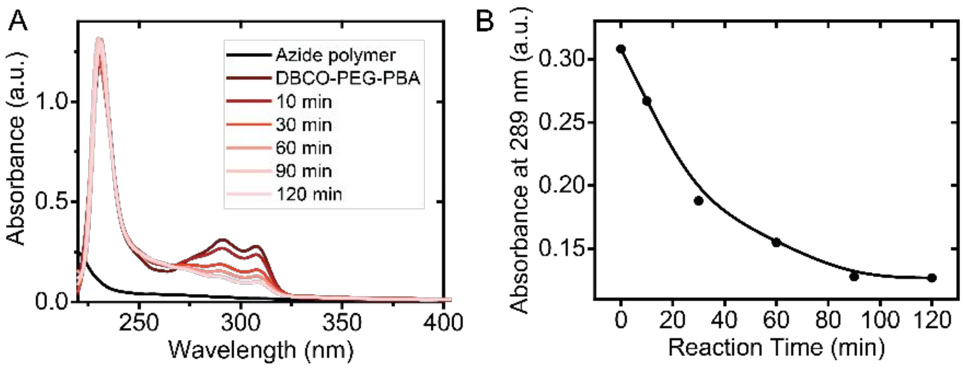

Figure S1. UV-Vis monitoring of the reaction kinetics between PAcrAm-g-PEG-Azide and DBCO-PEG-PBA in solution. A. UV absorption spectra of solutions containing 0.1 mg/mL PAcrAm-g-PEG-Azide only (black), 20 mg/mL DBCO-PEG-PBA only, and their mixture with 0.1 mg/mL PAcrAm-g-PEG-Azide and 20 mg/mL DBCO-PEG-PBA after increasing reaction times. **B.** Decrease of the characteristic UV absorbance peak of the DBCO group at 289 nm over time, indicating the progression of the reaction. The reaction reached a plateau after 90 min; we chose a shorter 60 min incubation time for nanopore coating to minimize the risk of pore clogging.

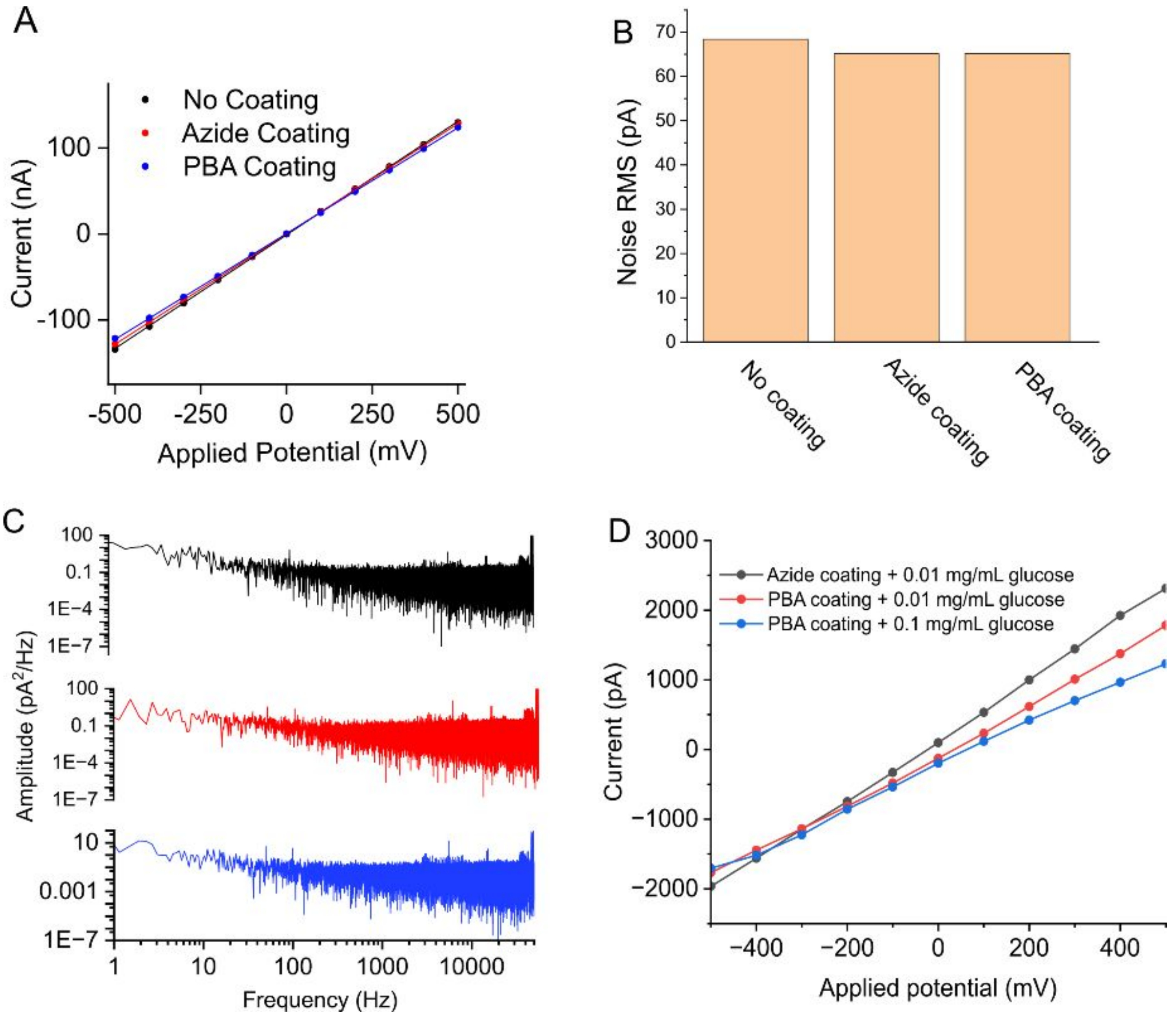

Figure S2. Effects of different nanopore coatings on the current-voltage relationship, noise, and power density spectrum from these pores. A. Current-Voltage curves of bare (black), Azide-coated (red), and PBA-coated (blue) nanopores. The measurements were performed using a nanopore with a diameter of 18 nm in a recording electrolyte containing 2 M KCl, 10 mM HEPES, pH 7.4. **B.** Root mean square of the baseline noise at applied potential of 100 mV and a bandwidth of ~ 57 kHz. **C.** Power density spectrum of bare (black), Azide-coated (red), and PBA-coated (blue) nanopores. **D.** Current-Voltage curves of Azide-coated nanopore in the presence of 0.01 mg/mL glucose (black), with a slope of 4.5 nA/V, PBA-coated nanopore in the presence of 0.01 mg/mL glucose (red)), with a slope of 3.2 nA/V, and PBA-coated nanopore in the presence of 0.1 mg/mL glucose (blue)), with a slope of 2.9 nA/V.

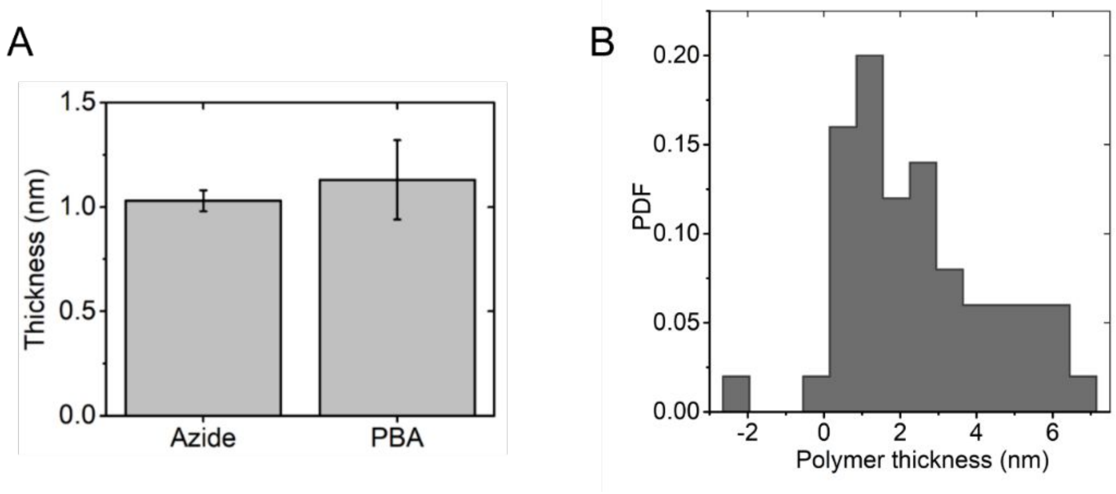

Figure S3. Analysis of the thickness of PAcrAm-g-PEG-Azide and PAcrAm-g-PEG-PBA coatings on SiN_x substrates by ellipsometry. A. Ellipsometry measurements of a dried PAcrAm-g-PEG-PBA coating and a PAcrAm-g-PEG-Azide coating. The substrate used for coating consisted of three layers: bulk Si, 110 nm of SiO_2, and a 30 nm top layer of Si_3N_4. **B.** Histogram of the measured thickness of PAcrAm-g-PEG-PBA coating by ion conductance from nanopores of different diameters ranging from 10 to 25 nm. The average thickness and standard deviation of the PAcrAm-g-PEG-PBA coating are 1.4 ± 1.3 nm (N = 50).

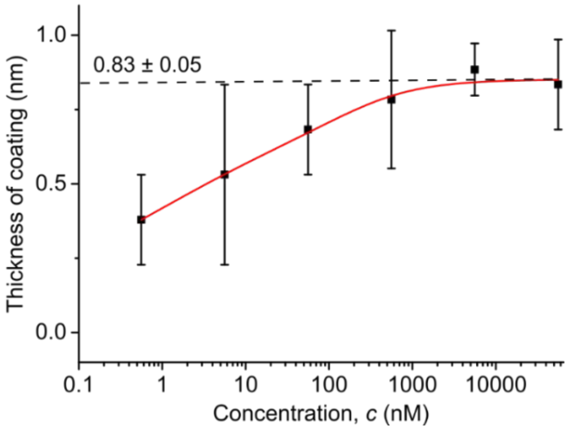

Figure S4. Effective thickness of the glucose layer as a function of glucose concentration in a PBA-coated nanopore. The dashed line indicates the maximum thickness of the glucose layer (0.83 ± 0.05 nm). The red curve shows the results of fitting a two-site Langmuir isotherm (Equation S3) to the coating thickness as a function of glucose concentration. Ionic current through the nanopore was recorded at -100 mV applied voltage with 500 kHz sampling rate in 2 M KCl, 10 mM HEPES, pH 7.5, at various glucose concentrations. Error bars represent the standard deviation of three independent measurements. The fitting yielded an adjusted R^2 of 0.99, maximum adsorption capacities of 0.32 and 0.53 nm, and dissociation constants of 71.49 and 0.235 nM. Note that the highest glucose concentration used in this experiment was 10 g/L (56 mM), and the corresponding increase in bulk viscosity of the recording electrolyte reduced its conductivity by less than 3%. This reduction is equivalent to a maximum error in coating thickness of 0.1 nm and therefore very small.

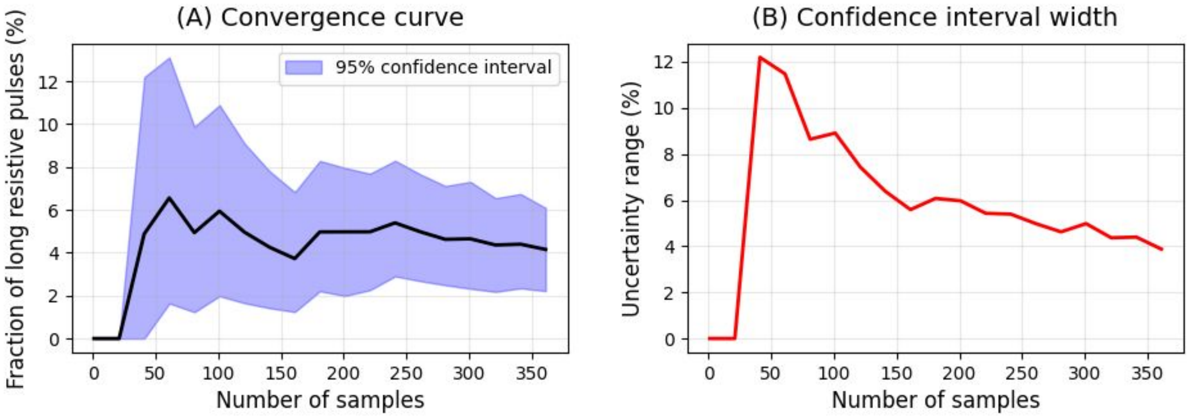

Figure S5. Validation of the conclusions drawn from the results shown in Figure 2 A as a function of the sample size. The median fraction of long resistive pulses ($t_d > 0.4$ ms, solid blue line) and the corresponding 95% confidence interval converge to 4 ± 2% as the sample size increases, demonstrating the stability of the distribution and hence an adequate sample size. This analysis is based on a dataset with a sample size of 378, utilizing bootstrap resampling with 1000 repetitions. **B.** The uncertainty of the fraction of long resistive pulses reaches a value below 6% after approximately 200 samples are collected, and below 5% after 280 samples, confirming that the sample size was sufficient to support the conclusion.

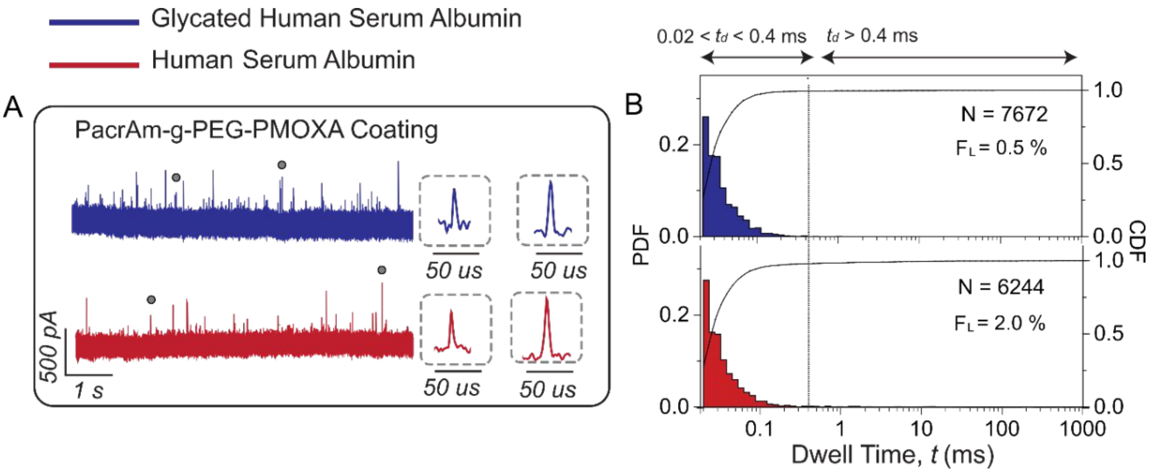

Figure S6. Control experiment showing recordings of translocations of gHSA and HSA through PAcrAm-g-PEG-PMOXA-coated SiNx nanopore; these protein-resistant coating did not present a PBA group. A) Representative ionic current recordings of HSA (red) and gHSA (blue) in PMOXA-coated nanopores. Insets display individual resistive pulses **B)** Probability density functions (PDF) and cumulative distribution functions (CDF) of the logarithm of dwell times from the translocation of gHSA (blue) or HSA (red). Dotted line represents the dwell time threshold of 400 µs for determining the long trapping events and free translocation events. All events that have a dwell time of at least 20 µs and are filtered with a cutoff frequency of 50 kHz with a digital Gaussian low-pass filter. This experiment reveals that coatings without PBA groups do not trap glycated (or unglycated) proteins as expected.

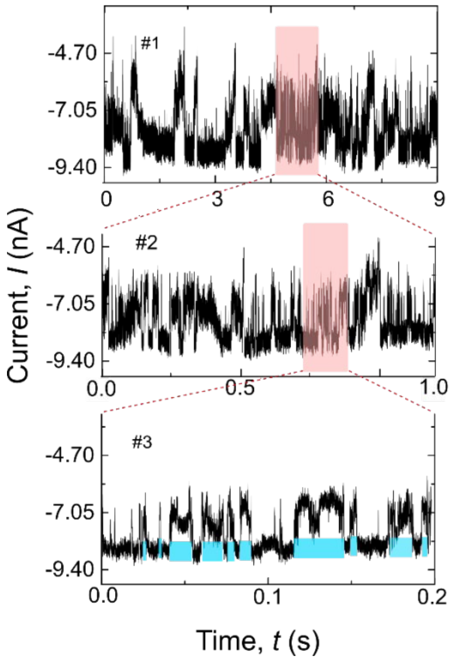

Figure S7. Sequential zoom-in views of a representative gHSA current trace shown in Figure 2E highlighting finer temporal features at increasing resolution. Areas shaded in red indicate the zoomed-in sections.

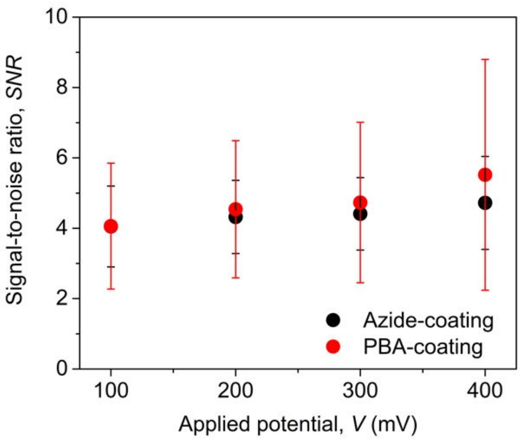

Figure S8. Signal-to-noise ratio of ionic current obtained in the presence of gHSA as a function of the applied potential difference. The signal-to-noise ratio for each individual resistive pulse was calculated as the ratio of the average pulse amplitude to the standard deviation of the local baseline. Only resistive pulses with dwell time of at least 20 μs were included in the analysis.

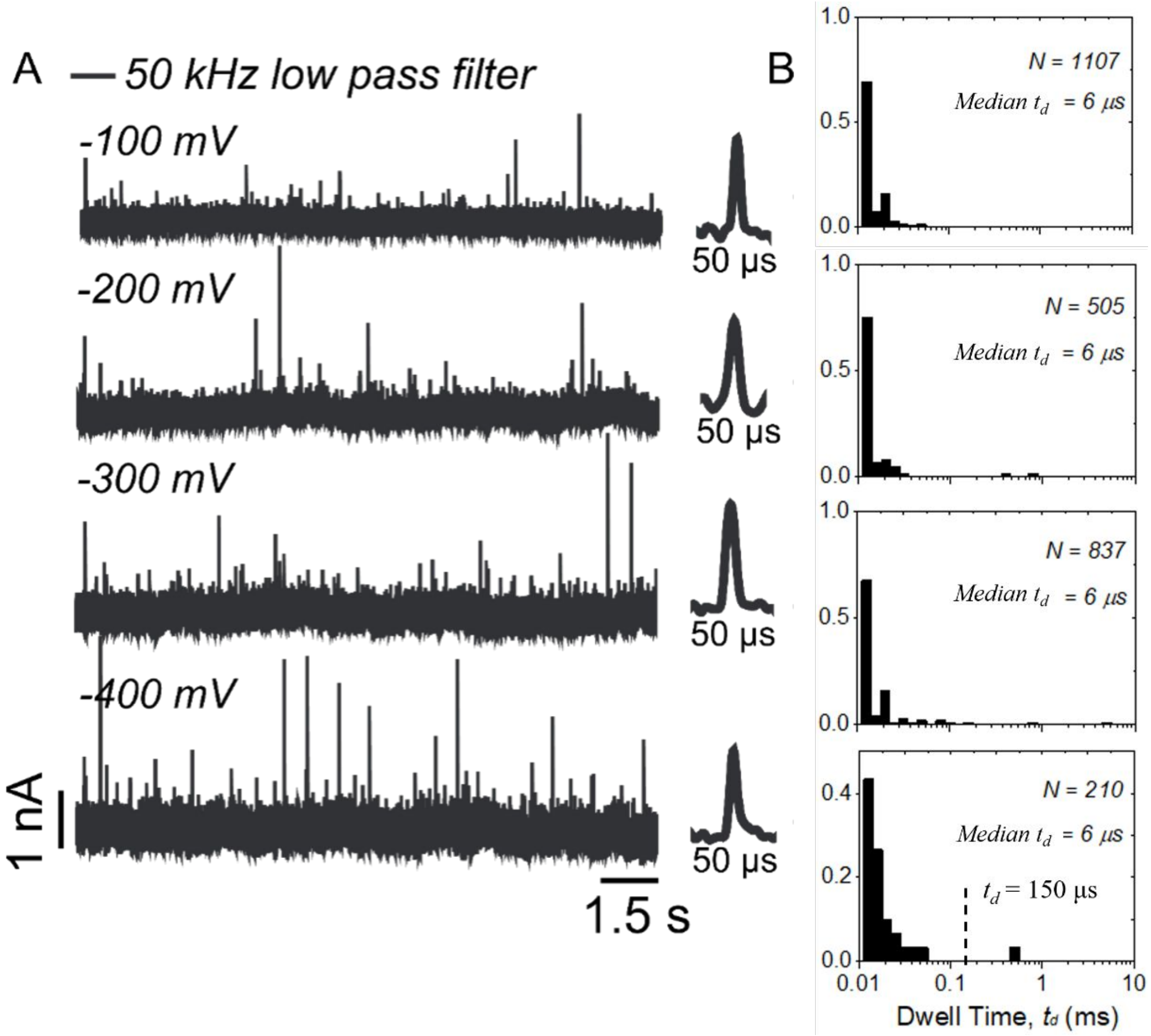

Figure S9. Control experiment: Analysis of dwell times of gHSA in PAcrAm-g-PEG-Azide-coated nanopores at different applied voltages. A. Representative current traces at different applied voltages, Gaussian low-pass filtered at 50 kHz. **B.** Corresponding PDFs of dwell times at different applied voltages. The median dwell time, t_d, was calculated without applying any dwell time thresholds. The dashed line at t_d = 150 µs indicates the minimum dwell time required for volume and shape analysis. All measurements were performed with the same nanopore as in **Figure 4.**

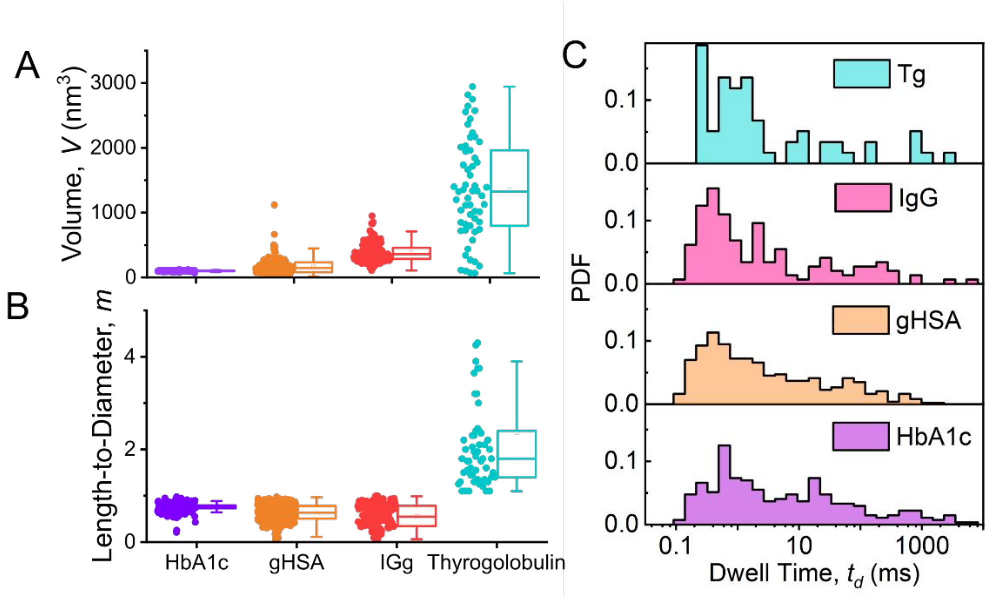

Figure S10. Analysis of the volume and shape of four individual natively folded proteins based on resistive pulses in PBA-coated nanopores. (A) Estimated excluded volume and **(B)** length-to-diameter ratio of four test proteins. **C.** Probability density function (PDF) of dwell times of those four test proteins. Data were collected using nanopores of different diameters as explained in the main text, at a 500 kHz sampling rate, and filtered with a 50 kHz Gaussian low-pass filter.

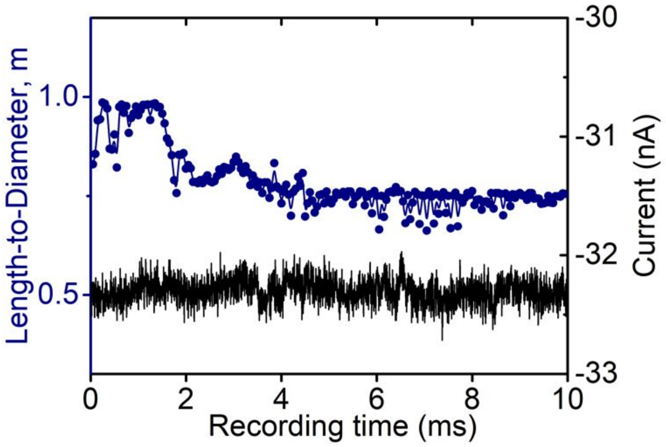

Figure S11. The precision of the estimation of length-to-diameter ratio estimation based on a single event improves with increasing residence time of the protein in the nanopore. The black curve is a representative current trace obtained with a PBA-coated nanopore in the presence of gHSA and Gaussian low-pass filtered at 50 kHz. The blue curve displays the results of length-to-diameter ratio estimation based on this trace as a function of increasing residence time t_d in the nanopore in the range from 0 to 10 ms.

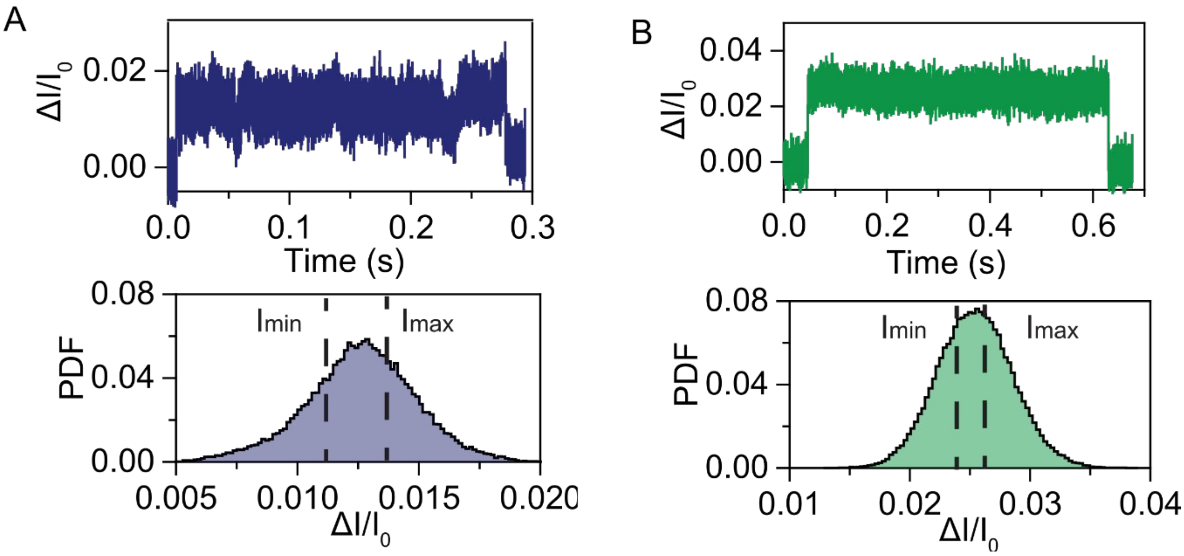

Figure S12. Shape and volume estimation of two glycated proteins, gHSA and HbA1c, with PBA-coated nanopores. A, B: Examples of original current traces of long trapping events and corresponding histograms of relative blockade current, $\Delta I/I_0$, for (A) gHSA, and (B) HbA1c. The I_{min} and I_{max} were determined by fitting the probability density distribution of the relative blockade current with **Equation S8**. The experiments were performed at -200 mV applied voltage, 500 kHz sampling rate, 50 kHz Gaussian low-pass filter in 2 M KCl, 10 mM HEPES, pH 7.4 recording buffer.

References

1. Yusko, E. C.; Bruhn, B. R.; Eggenberger, O. M.; Houghtaling, J.; Rollings, R. C.; Walsh, N. C.; Nandivada, S.; Pindrus, M.; Hall, A. R.; Sept, D.; Li, J. L.; Kalonia, D. S.; Mayer, M., Real-Time Shape Approximation and Fingerprinting of Single Proteins Using a Nanopore. *Nat Nanotechnol* **2017,** *12*, 360-367.

2. Springsteen, G.; Wang, B. H., A Detailed Examination of Boronic Acid-Diol Complexation. *Tetrahedron* **2002,** *58*, 5291-5300.

3. Wei, R. S.; Gatterdam, V.; Wieneke, R.; Tampé, R.; Rant, U., Stochastic Sensing of Proteins with Receptor-Modified Solid-State Nanopores. *Nat Nanotechnol* **2012,** *7*, 257-263.

4. Ribas-Arino, J.; Shiga, M.; Marx, D., Mechanochemical Transduction of Externally Applied Forces to Mechanophores. *J Am Chem Soc* **2010,** *132*, 10609-10614.

5. Wei, R. S.; Gatterdam, V.; Wieneke, R.; Tampe, R.; Rant, U., Stochastic Sensing of Proteins with Receptor-Modified Solid-State Nanopores. *Nat Nanotechnol* **2012,** *7*, 257-263.

6. Awasthi, S.; Sriboonpeng, P.; Ying, C. F.; Houghtaling, J.; Shorubalko, I.; Marion, S.; Davis, S. J.; Sola, L.; Chiari, M.; Radenovic, A.; Mayer, M., Polymer Coatings to Minimize Protein Adsorption in Solid-State Nanopores. *Small Methods* **2020,** *4*, 2000177.

7. Pedone, D.; Firnkes, M.; Rant, U., Data Analysis of Translocation Events in Nanopore Experiments. *Anal Chem* **2009,** *81*, 9689-9694.

8. Houghtaling, J.; Ying, C. F.; Eggenberger, O. M.; Fennouri, A.; Nandivada, S.; Acharjee, M.; Li, J. L.; Hall, A. R.; Mayer, M., Estimation of Shape, Volume, and Dipole Moment of Individual Proteins Freely Transiting a Synthetic Nanopore. *Acs Nano* **2019,** *13*, 5231-5242.

9. Yusko, E. C.; Johnson, J. M.; Majd, S.; Prangkio, P.; Rollings, R. C.; Li, J. L.; Yang, J.; Mayer, M., Controlling Protein Translocation through Nanopores with Bio-Inspired Fluid Walls. *Nat Nanotechnol* **2011,** *6*, 253-260.

10. Bowman, N.; Heath, M. T., Computing Minimum-Volume Enclosing Ellipsoids. *Math Program Comput* **2023,** *15*, 621-650.

11. Richards, F. M., Areas, Volumes, Packing, and Protein-Structure. *Annu Rev Biophys Bio* **1977,** *6*, 151-176.

12. Chothia, C., Structural Invariants in Protein Folding. *Nature* **1975,** *254*, 304-308.
